# Crystal structure of bis­(piperazin-1-ium-κ*N* ^4^)bis­(thio­sulfato-κ*S*)zinc(II) dihydrate

**DOI:** 10.1107/S2056989018000555

**Published:** 2018-01-19

**Authors:** Avijit Kumar Paul

**Affiliations:** a Department of Chemistry, National Institute of Technology Kurukshetra, Haryana 136 119, India

**Keywords:** crystal structure, zinc thio­sulfate, piperazine, hydrogen bonding

## Abstract

In the title compound, [Zn(C_4_H_11_N_2_)_2_(S_2_O_3_)_2_]·2H_2_O, two thio­sulfate ions coordinate to the zinc(II) atom through the terminal S atoms. The tetra­hedral coordination around the Zn^II^ ion is completed by ligating to two N atoms of two piperazinium ions. The remaining two N atoms of the piperazinium ions are diprotonated and do not coordinate to the metal centre. In the crystal, however, they are involved in N—H⋯O_water_ and N—H⋯Osulfato hydrogen bonds. Together, a series of N—H⋯O and O—H⋯O hydrogen bonds, involving the O atoms of the thio­sulfate ions and the water mol­ecules as acceptors and the hydrogen atoms of the piperazinium ions and the water mol­ecules as donors, form a three-dimensional supra­molecuar structure. Within this framework there are a number of intra- and inter­molecular C—H⋯O and C—H⋯S contacts present.

## Abstract 2

In the title compound, two thio­sulfate ions coordinate to the zinc(II) ion through the terminal S atoms. The tetra­hedral coordination around the Zn^II^ ion is completed by the ligation of two N atoms of two piperazinium ions. In the crystal, a network of N—H⋯O and O—H⋯O hydrogen bonds leads to the formation of a three-dimensional supra­molecular structure.

## Chemical context   

Over the last few decades, a large number of amine-templated metal complexes and compounds with extended structures have been synthesized in the presence of a number of inorganic anions (Férey, 2008 ▸). One series of anions, namely the sulfur-containing oxoanions, and in particular sulfates and sulfites, are widely used in the synthesis of higher dimensional inorganic compounds because of their multidentate coordin­ation capacity towards metal ions (Rao *et al.*, 2006 ▸). In these examples, the anions bind to the metal cations through the oxygen atoms. The thio­sulfate ion is a new example of an sulfur oxoanion used in amine-templated synthesis, although the reactivity of this ligand is less than that of the sulfate and sulfite ions. In this heteroatomic ligand, the terminal S atom, as well as the O atoms, can bind to a range of metal ions. However, the long S—S bond is unstable under acidic conditions or at high temperature. Hence, the thio­sulfate anion has not, to date, been explored extensively as a network-building unit for higher dimensional structures (Paul *et al.*, 2011 ▸). Despite these stability complications, Baggio and co-workers have synthesized a few mol­ecular and one-dimensional structures containing thio­sulfate anions that are connected to the metal through oxygen as well as sulfur atoms (Baggio *et al.*, 1996 ▸, 1997 ▸; Freire *et al.*, 2001 ▸; Harvey *et al.*, 2004 ▸). Our continuing synthetic efforts using the thio­sulfate anion have resulted in the synthesis of some new three-dimensional structures in the family of cadmium–thio­sulfate hybrid compounds formed in the presence of organic linkers (Paul *et al.*, 2009*a* ▸,*b* ▸, 2010 ▸). It is noteworthy that all of the reported metal–thio­sulfate compounds are synthesized in the presence of nitro­gen-containing aromatic organic linkers. Aromatic ligands play a dual role in metal–thio­sulfate formation as they increase the dimensionality of the local structure and increase structure stabilization *via* secondary inter­actions, such as hydrogen bonds. Recently, Natarajan and co-workers (Karthik & Natarajan, 2016 ▸) have reported on some three-dimensional zinc–thio­sulfate hybrid structures with aromatic N-donor organic linkers. Metal–thio­sulfate compounds prepared in the presence of aliphatic amines are, however, rare (Paul, 2016 ▸) and require investigation. The title compound, is the first example of an aliphatic-amine-templated zinc thio­sulfate compound. Its synthesis and crystal structure are reported on herein.
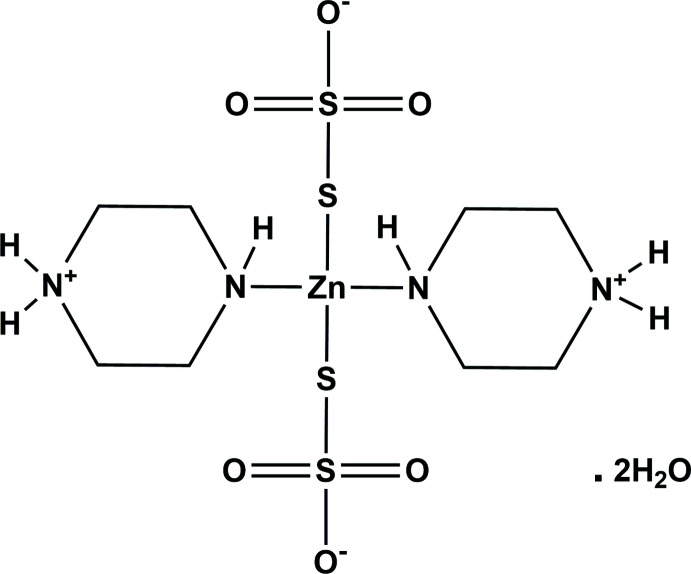


## Structural commentary   

**Figure 1:**
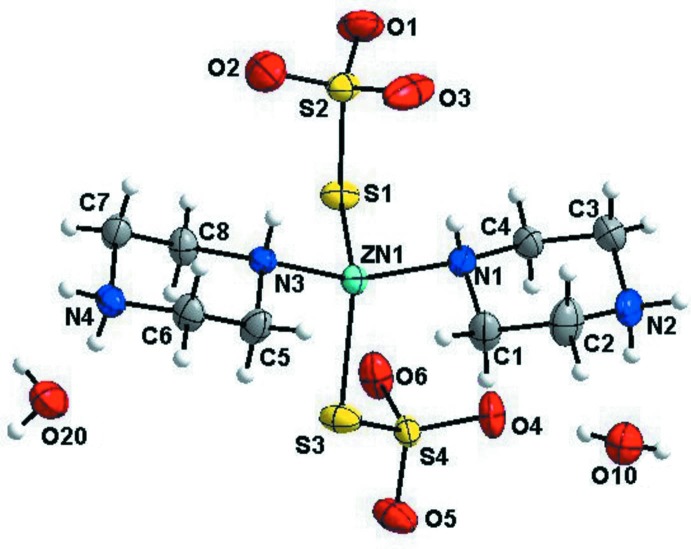
The asymmetric unit of the title compound, with atom labelling and showing 50% probability displacement ellipsoids.

The mol­ecular structure of the title compound is illustrated in Fig. 1 ▸. In the complex, the Zn^2+^ ion is coordinated by two sulfur atoms of the thio­sulfate ligands (S1 and S3) and two nitro­gen atoms from the piperazinium ions (N1 and N3), in an approximately tetra­hedral geometry (ZnS_2_N_2_, CN = 4). The Zn—S bond lengths are 2.2927 (4) Å for Zn1—S1 and 2.3324 (4) Å for Zn1—S3. The Zn—N bond lengths are 2.0879 (13) Å for Zn1—N1 and 2.0727 (12) Å for Zn1—N3. The N/S—Zn1—S/N bond angles lie in the range 101.24 (4) to 116.79 (2)°, confirming the tetra­hedral nature of the zinc ions. Within the two thio­sulfate ligands, the S—S bond lengths are 2.0511 (5) Å for S1—S2 and 2.0332 (5) Å for S3—S4. The S—O bond lengths vary from 1.4437 (14) to 1.4623 (13) Å, while the O—S—O angles vary from 104.53 (5) to 112.85 (10)°, which is indicative of a fairly regular tetra­hedral arrangement. In the mol­ecular unit, the two thio­sulfate units are bonded to the zinc(II) ion only through the terminal S atoms, and the oxygen atoms are uncoordinated. In addition, only one nitro­gen atom of each piperazinium ion is bonded to the zinc(II) ion, the second being diprotonated in each case.

## Supra­molecular features   

**Figure 2:**
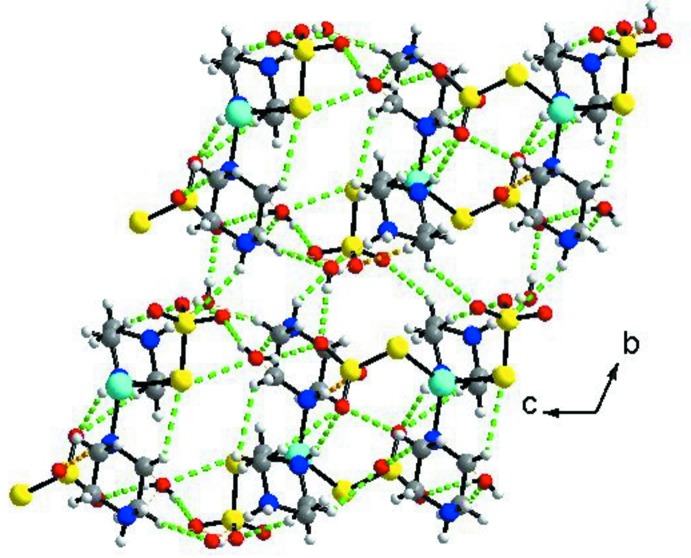
A view along the *a* axis of the crystal packing of the title compound. The hydrogen bonds are shown as dashed lines (see Table 1 ▸).

The supra­molecular architecture (Fig. 2 ▸) arises from a three-dimensional network of N—H⋯O and O—H⋯O hydrogen bonds involving the uncoordinated oxygen atoms of the thio­sulfate ligands, the protonated piperazine units and the lattice water mol­ecules (Table 1 ▸). These inter­molecular inter­actions lead to the formation of a supra­molecular framework. Within this framework there are a number of intra- and inter­molecular C—H⋯O and C—H⋯S contacts present (Table 1 ▸).

## Database survey   

A search of the Cambridge Structural Database (CSD, Version 35.9, last update May 2017; Groom *et al.*, 2016 ▸) for zinc–thio­sulfato complexes gave 12 hits, all involving aromatic amines and/or thio­ureas. Díaz de Vivar *et al.* (2006 ▸) have described a mol­ecular zinc–thio­sulfate complex prepared in the presence of a tridentate aromatic ligand, *viz*. aqua(thio­sulfato-κ*O*,*S*)[2,4,6-tris­(2-pyrid­yl)-1,3,5-triazine-*N*,*N*′,*N*′′]zinc(II) hemihydrate (CSD refcode: WEHTOT). The thio­sulfate ligand is coordinated to the zinc ions through S and O atoms, forming octa­hedral zinc centres. In addition, a zinc–thio­sulfate complex containing both one-dimensional cationic and anionic chains has been reported by the same authors, *viz. catena*-[(μ^2^-4,4′-bi­pyridine-κ*N*,*N*′)tetra­aqua­zinc(II) bis­(μ^2^-4,4′-bi­pyridine-κ*N*,*N*′)(μ^2^-thio­sulfato-κ*O*,*S*)bis­(thio­sulfato-κ*S*)dizinc(II) dihydrate] [PEYLEL; Díaz de Vivar *et al.*, 2007 ▸). Both types of chain contain 4,4′-bi­pyridine ligands as linkers.

Karthik & Natarajan (2016 ▸) have recently reported four higher-dimensional zinc–thio­sulfate compounds synthesized in the presence of various aromatic ligands, *viz. catena*-[bis­(μ-4,4′-bi­pyridine)­bis­(μ-thio­sulfato)­dizinc] (IJUWER), *catena*-[(μ-4,4′-propane-1,3-diyldi­pyridine)(μ-thio­sulfato)­zinc] (IJU­WIV), and *catena*-[bis­(μ-4,4′-ethene-1,2-diyldi­pyridine)­bis­(μ-thio­sulfato)­dizinc dihydrate] (IJUWOB) and *catena*-[bis­(μ-4,4′-ethane-1,2-diyldi­pyridine)­bis­(μ-thio­sulfato)­dizinc (μ-4,4′-ethane-1,2-diyldi­pyridine)(μ-thio­sulfato)­zinc trihydrate] (IJUWUH).

A number of mol­ecular cadmium–thio­sulfate and manganese–thio­sulfate structures have been reported by Baggio and co-workers (Baggio *et al.*, 1996 ▸, 1997 ▸; Freire *et al.*, 2001 ▸; Harvey *et al.*, 2004 ▸). They were synthesized in the presence of 2,2′-bi­pyridine or 1,10-phenanthroline.

There are a few examples in which zero-dimensional cadmium–thio­sulfate compounds form simple dinuclear complexes, in which the thio­sulfate unit is bound to the metal through both the sulfur and the oxygen atoms. As expected, the structures are stabilized through C—H⋯O hydrogen-bonding inter­actions and π–π inter­actions. One cadmium thio­sulfate compound, bis­(propane-1,3-di­amine)(thio­sulfato)cadmium (CSD refcode: ORUJOC), which was reported recently, was isolated in the presence of the aliphatic amine 1,3-di­amino­propane (Paul, 2016 ▸). One mol­ecular piperazinium thio­sulfate monohydrate structure has been reported, (piperazinediium thio­sulfate monohydrate; CSD refcode: AROWUA; Srinivasan *et al.*, 2011 ▸), in which the protonated aliphatic amine and thio­sulfate units are linked together through extensive hydrogen bonds. It is noteworthy that there are no previous examples in the literature of zinc–thio­sulfate structures that crystallize in the presence of aliphatic amines.

## Synthesis and crystallization   

Zn(NO_3_)_2_·6H_2_O (0.297 g, 1 mmol) was dissolved in 5 ml distilled water. Then (NH_4_)_2_S_2_O_3_ (0.296 g, 2 mmol) was added to the solution, which was stirred for 15 min. Piperazine (0.172 g, 2 mmol) was dissolved separately in distilled water (5 ml) and the solution poured into the initial reaction mixture until the pH was 8. The resulting solution was left undisturbed and after 1 week, colourless block-shaped crystals were obtained. The product was filtered and washed with cold water. The yield was approximately 85% based on Zn metal. Elemental analysis calculated for C_8_H_26_N_4_O_8_S_4_Zn: C 19.20, H 5.24, N 11.20%; found: C 19.27, H 5.29, N 11.16%.

## Refinement   

Crystal data, data collection and structure refinement details are summarized in Table 2 ▸. The NH, NH_2_ and water H atoms were located in difference-Fourier maps and freely refined. The C-bound H atoms were included in calculated positions and refined as riding: C—H = 0.97 Å with *U*_iso_(H) = 1.2*U*_eq_(C).

## Supplementary Material

Crystal structure: contains datablock(s) I, Global. DOI: 10.1107/S2056989018000555/cq2022sup1.cif

Structure factors: contains datablock(s) I. DOI: 10.1107/S2056989018000555/cq2022Isup2.hkl

CCDC reference: 1571150

Additional supporting information:  crystallographic information; 3D view; checkCIF report

## Figures and Tables

**Table 1:** Hydrogen-bond geometry (Å, °)

*D*—H⋯*A*	*D*—H	H⋯*A*	*D*⋯*A*	*D*—H⋯*A*
N1—H1⋯O2^i^	0.83 (2)	2.25 (2)	3.041 (2)	160 (2)
N2—H2*AN*⋯O6^ii^	0.95 (3)	1.80 (3)	2.730 (2)	166 (2)
N2—H2*BN*⋯O10	0.93 (2)	1.89 (2)	2.811 (3)	170 (2)
N3—H3⋯O3^i^	0.84 (2)	2.03 (2)	2.853 (2)	166 (2)
N4—H4*AN*⋯O20	0.87 (2)	2.09 (2)	2.892 (2)	154 (2)
N4—H4*BN*⋯O5^iii^	0.94 (2)	1.84 (2)	2.763 (2)	169 (2)
O10—H10*A*⋯O4	0.85 (3)	1.94 (4)	2.780 (2)	175 (3)
O10—H10*B*⋯O1^iv^	0.76 (3)	2.02 (3)	2.777 (2)	176 (4)
O20—H20*A*⋯O1^v^	0.82 (3)	2.02 (3)	2.804 (2)	163 (3)
O20—H20*B*⋯O5^vi^	0.74 (3)	2.17 (3)	2.866 (2)	158 (3)
C3—H3*A*⋯O6^iv^	0.97	2.45	3.221 (2)	136
C4—H4*A*⋯O3	0.97	2.49	3.175 (3)	128
C4—H4*B*⋯O4	0.97	2.54	3.463 (2)	159
C5—H5*B*⋯S3	0.97	2.86	3.453 (2)	120
C8—H8*B*⋯O2	0.97	2.50	3.318 (2)	142

Symmetry codes: (i) 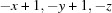; (ii) 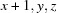; (iii) 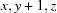; (iv) 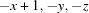; (v) 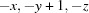; (vi) 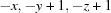.

**Table 2:** Experimental details

Crystal data	
Chemical formula	[Zn(C_4_H_11_N_2_)_2_(S_2_O_3_)_2_]·2H_2_O
*M*_r_	499.94
Crystal system, space group	Triclinic, *P* 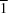
Temperature (K)	293
*a*, *b*, *c* (Å)	8.7631 (1), 10.5623 (2), 11.6072 (2)
α, β, γ (°)	113.736 (1), 98.761 (1), 91.472 (1)
*V* (Å^3^)	967.49 (3)
*Z*	2
Radiation type	Mo *K*α
μ (mm^−1^)	1.74
Crystal size (mm)	0.22 × 0.18 × 0.16

Data collection	
Diffractometer	Bruker SMART APEX CCD area detector
Absorption correction	Multi-scan (*SADABS*; Bruker, 2000 ▸)
*T*_min_, *T*_max_	0.700, 0.768
No. of measured, independent and observed [*I* > 2σ(*I*)] reflections	19938, 7607, 5927
*R*_int_	0.027
(sin θ/λ)_max_ (Å^−1^)	0.782

Refinement	
*R*[*F* ^2^ > 2σ(*F* ^2^)], *wR*(*F* ^2^), *S*	0.032, 0.079, 1.01
No. of reflections	7607
No. of parameters	266
H-atom treatment	H atoms treated by a mixture of independent and constrained refinement
Δρ_max_, Δρ_min_ (e Å^−3^)	0.47, −0.36

Computer programs: *SMART* and *SAINT* (Bruker, 2000 ▸), *SHELXS97* (Sheldrick, 2008 ▸), *SHELXL2016/6* (Sheldrick, 2015 ▸), *ORTEP-3 for Windows* (Farrugia, 2012 ▸), *PLATON* (Spek, 2009 ▸) and *publCIF* (Westrip, 2010 ▸).

## supplementary crystallographic information

### Crystal data

**Table:**

[Zn(C_4_H_11_N_2_)_2_(S_2_O_3_)_2_]·2H_2_O	*Z* = 2
*M**_r_* = 499.94	*F*(000) = 520
Triclinic, *P*1	*D*_x_ = 1.716 Mg m^−^^3^
*a* = 8.7631 (1) Å	Mo *K*α radiation, λ = 0.71073 Å
*b* = 10.5623 (2) Å	Cell parameters from 3790 reflections
*c* = 11.6072 (2) Å	θ = 2.0–26.0°
α = 113.736 (1)°	µ = 1.74 mm^−^^1^
β = 98.761 (1)°	*T* = 293 K
γ = 91.472 (1)°	Block, colorless
*V* = 967.49 (3) Å^3^	0.22 × 0.18 × 0.16 mm

### Data collection

**Table:**

Bruker SMART APEX CCD area detector diffractometer	7607 independent reflections
Radiation source: fine-focus sealed tube	5927 reflections with *I* > 2σ(*I*)
Graphite monochromator	*R*_int_ = 0.027
φ and ω scans	θ_max_ = 33.7°, θ_min_ = 2.0°
Absorption correction: multi-scan (SADABS; Bruker, 2000)	*h* = −13→13
*T*_min_ = 0.700, *T*_max_ = 0.768	*k* = −16→9
19938 measured reflections	*l* = −18→17

### Refinement

**Table:**

Refinement on *F*^2^	Primary atom site location: structure-invariant direct methods
Least-squares matrix: full	Secondary atom site location: difference Fourier map
*R*[*F*^2^ > 2σ(*F*^2^)] = 0.032	Hydrogen site location: mixed
*wR*(*F*^2^) = 0.079	H atoms treated by a mixture of independent and constrained refinement
*S* = 1.01	*w* = 1/[σ^2^(*F*_o_^2^) + (0.040*P*)^2^] where *P* = (*F*_o_^2^ + 2*F*_c_^2^)/3
7607 reflections	(Δ/σ)_max_ < 0.001
266 parameters	Δρ_max_ = 0.47 e Å^−^^3^
0 restraints	Δρ_min_ = −0.36 e Å^−^^3^

### Special details

**Table:**

Geometry. All esds (except the esd in the dihedral angle between two l.s. planes) are estimated using the full covariance matrix. The cell esds are taken into account individually in the estimation of esds in distances, angles and torsion angles; correlations between esds in cell parameters are only used when they are defined by crystal symmetry. An approximate (isotropic) treatment of cell esds is used for estimating esds involving l.s. planes.

### Fractional atomic coordinates and isotropic or equivalent isotropic displacement parameters (Å^2^)

**Table:**

	*x*	*y*	*z*	*U*_iso_*/*U*_eq_	
Zn1	0.36403 (2)	0.36678 (2)	0.17364 (2)	0.02307 (5)	
S1	0.19769 (4)	0.23705 (4)	−0.01347 (4)	0.02921 (8)	
S2	0.28149 (4)	0.31755 (4)	−0.12806 (4)	0.02866 (8)	
S3	0.31486 (6)	0.34577 (4)	0.35739 (4)	0.03543 (10)	
S4	0.27051 (4)	0.13632 (4)	0.29154 (4)	0.02567 (8)	
O1	0.22237 (19)	0.21735 (14)	−0.25891 (12)	0.0508 (4)	
O2	0.22392 (19)	0.45077 (14)	−0.10572 (15)	0.0541 (4)	
O3	0.44978 (15)	0.32601 (19)	−0.09909 (15)	0.0612 (4)	
O4	0.40860 (15)	0.06807 (14)	0.25701 (14)	0.0486 (3)	
O5	0.2234 (2)	0.11500 (13)	0.39847 (14)	0.0544 (4)	
O6	0.14635 (14)	0.09045 (13)	0.18082 (13)	0.0435 (3)	
N1	0.59630 (14)	0.33597 (13)	0.16071 (13)	0.0261 (2)	
H1	0.627 (2)	0.3885 (19)	0.1297 (18)	0.033 (5)*	
N2	0.86568 (17)	0.19550 (17)	0.20334 (16)	0.0381 (3)	
H2AN	0.968 (3)	0.172 (2)	0.194 (2)	0.053 (6)*	
H2BN	0.831 (3)	0.149 (2)	0.249 (2)	0.058 (7)*	
N3	0.34859 (14)	0.57724 (12)	0.22385 (12)	0.0225 (2)	
H3	0.396 (2)	0.5979 (17)	0.1754 (17)	0.024 (4)*	
N4	0.24360 (17)	0.84404 (13)	0.37115 (13)	0.0298 (3)	
H4AN	0.186 (2)	0.8219 (19)	0.4161 (19)	0.037 (5)*	
H4BN	0.229 (2)	0.937 (2)	0.3879 (19)	0.043 (5)*	
C1	0.69716 (19)	0.37986 (18)	0.28807 (16)	0.0381 (4)	
H1A	0.654430	0.334114	0.335305	0.046*	
H1B	0.696156	0.479233	0.335289	0.046*	
C2	0.8636 (2)	0.34663 (19)	0.2805 (2)	0.0454 (5)	
H2A	0.911324	0.399270	0.241316	0.055*	
H2B	0.922320	0.372326	0.365901	0.055*	
C3	0.7752 (2)	0.1542 (2)	0.07222 (18)	0.0421 (4)	
H3A	0.777441	0.055408	0.022780	0.051*	
H3B	0.821264	0.203770	0.029594	0.051*	
C4	0.60942 (19)	0.18742 (16)	0.07979 (15)	0.0315 (3)	
H4A	0.552976	0.164170	−0.005869	0.038*	
H4B	0.561198	0.129930	0.114233	0.038*	
C5	0.4203 (2)	0.66344 (15)	0.35818 (15)	0.0327 (3)	
H5A	0.528902	0.647499	0.370824	0.039*	
H5B	0.370312	0.634514	0.413833	0.039*	
C6	0.40821 (19)	0.81761 (15)	0.39612 (16)	0.0330 (3)	
H6A	0.450934	0.868622	0.486215	0.040*	
H6B	0.467790	0.849836	0.347563	0.040*	
C7	0.17408 (19)	0.76190 (16)	0.23399 (16)	0.0333 (3)	
H7A	0.227849	0.791358	0.180529	0.040*	
H7B	0.065948	0.778454	0.219288	0.040*	
C8	0.18624 (17)	0.60873 (15)	0.19857 (16)	0.0308 (3)	
H8A	0.124751	0.578258	0.247102	0.037*	
H8B	0.143694	0.557112	0.108512	0.037*	
O10	0.72416 (18)	0.05969 (17)	0.32966 (15)	0.0460 (3)	
H10A	0.629 (4)	0.060 (3)	0.303 (3)	0.098 (11)*	
H10B	0.742 (3)	−0.015 (3)	0.313 (3)	0.074 (10)*	
O20	−0.01440 (19)	0.7318 (2)	0.44092 (18)	0.0557 (4)	
H20A	−0.082 (4)	0.729 (3)	0.383 (3)	0.092 (11)*	
H20B	−0.048 (3)	0.776 (3)	0.497 (3)	0.070 (9)*	

### Atomic displacement parameters (Å^2^)

**Table:**

	*U*^11^	*U*^22^	*U*^33^	*U*^12^	*U*^13^	*U*^23^
Zn1	0.02461 (8)	0.02117 (8)	0.02345 (8)	0.00381 (6)	0.00452 (6)	0.00900 (6)
S1	0.02902 (18)	0.03198 (19)	0.02573 (17)	−0.00689 (14)	0.00212 (13)	0.01250 (15)
S2	0.02756 (17)	0.0354 (2)	0.02670 (18)	0.00130 (14)	0.00454 (13)	0.01668 (15)
S3	0.0560 (3)	0.02537 (18)	0.02544 (18)	−0.00262 (17)	0.01059 (17)	0.01015 (15)
S4	0.02671 (16)	0.02414 (17)	0.03090 (18)	0.00813 (13)	0.01197 (13)	0.01342 (14)
O1	0.0746 (10)	0.0502 (8)	0.0237 (6)	0.0060 (7)	0.0030 (6)	0.0132 (5)
O2	0.0744 (10)	0.0403 (7)	0.0630 (9)	0.0151 (7)	0.0259 (8)	0.0316 (7)
O3	0.0291 (6)	0.1148 (13)	0.0692 (10)	−0.0021 (7)	0.0074 (6)	0.0689 (10)
O4	0.0356 (7)	0.0524 (8)	0.0618 (9)	0.0251 (6)	0.0179 (6)	0.0229 (7)
O5	0.0985 (12)	0.0319 (6)	0.0513 (8)	0.0170 (7)	0.0449 (8)	0.0242 (6)
O6	0.0308 (6)	0.0364 (7)	0.0466 (7)	0.0042 (5)	0.0010 (5)	0.0021 (5)
N1	0.0255 (6)	0.0260 (6)	0.0290 (6)	0.0060 (5)	0.0052 (5)	0.0132 (5)
N2	0.0270 (7)	0.0479 (9)	0.0476 (9)	0.0152 (6)	0.0120 (6)	0.0253 (7)
N3	0.0239 (5)	0.0217 (5)	0.0234 (6)	0.0036 (4)	0.0068 (4)	0.0097 (5)
N4	0.0379 (7)	0.0219 (6)	0.0331 (7)	0.0078 (5)	0.0141 (6)	0.0119 (5)
C1	0.0325 (8)	0.0364 (9)	0.0334 (8)	0.0085 (7)	−0.0011 (6)	0.0040 (7)
C2	0.0257 (8)	0.0450 (10)	0.0570 (12)	0.0036 (7)	−0.0024 (8)	0.0156 (9)
C3	0.0425 (9)	0.0500 (11)	0.0373 (9)	0.0219 (8)	0.0164 (7)	0.0172 (8)
C4	0.0335 (8)	0.0311 (8)	0.0273 (7)	0.0101 (6)	0.0061 (6)	0.0087 (6)
C5	0.0377 (8)	0.0255 (7)	0.0294 (7)	0.0066 (6)	−0.0024 (6)	0.0085 (6)
C6	0.0351 (8)	0.0231 (7)	0.0342 (8)	0.0025 (6)	0.0018 (6)	0.0065 (6)
C7	0.0349 (8)	0.0307 (8)	0.0336 (8)	0.0105 (6)	0.0027 (6)	0.0133 (6)
C8	0.0248 (7)	0.0269 (7)	0.0361 (8)	0.0044 (5)	0.0028 (6)	0.0091 (6)
O10	0.0428 (8)	0.0457 (8)	0.0492 (8)	0.0068 (6)	0.0067 (6)	0.0197 (7)
O20	0.0445 (8)	0.0857 (12)	0.0441 (9)	0.0215 (8)	0.0168 (7)	0.0300 (9)

### Geometric parameters (Å, º)

**Table:**

Zn1—N3	2.0727 (12)	N4—H4BN	0.93 (2)
Zn1—N1	2.0879 (13)	C1—C2	1.516 (2)
Zn1—S1	2.2927 (4)	C1—H1A	0.9700
Zn1—S3	2.3324 (4)	C1—H1B	0.9700
S1—S2	2.0511 (5)	C2—H2A	0.9700
S2—O2	1.4437 (14)	C2—H2B	0.9700
S2—O3	1.4539 (14)	C3—C4	1.510 (2)
S2—O1	1.4606 (13)	C3—H3A	0.9700
S3—S4	2.0332 (5)	C3—H3B	0.9700
S4—O4	1.4507 (12)	C4—H4A	0.9700
S4—O6	1.4546 (13)	C4—H4B	0.9700
S4—O5	1.4623 (13)	C5—C6	1.518 (2)
N1—C1	1.487 (2)	C5—H5A	0.9700
N1—C4	1.4876 (19)	C5—H5B	0.9700
N1—H1	0.83 (2)	C6—H6A	0.9700
N2—C2	1.485 (2)	C6—H6B	0.9700
N2—C3	1.489 (2)	C7—C8	1.511 (2)
N2—H2AN	0.95 (2)	C7—H7A	0.9700
N2—H2BN	0.93 (2)	C7—H7B	0.9700
N3—C5	1.4779 (19)	C8—H8A	0.9700
N3—C8	1.4820 (18)	C8—H8B	0.9700
N3—H3	0.837 (18)	O10—H10A	0.85 (3)
N4—C6	1.484 (2)	O10—H10B	0.76 (3)
N4—C7	1.491 (2)	O20—H20A	0.81 (3)
N4—H4AN	0.87 (2)	O20—H20B	0.74 (3)
			
N3—Zn1—N1	105.78 (5)	N1—C1—H1B	108.9
N3—Zn1—S1	110.79 (3)	C2—C1—H1B	108.9
N1—Zn1—S1	112.90 (4)	H1A—C1—H1B	107.7
N3—Zn1—S3	101.24 (4)	N2—C2—C1	109.22 (14)
N1—Zn1—S3	108.21 (4)	N2—C2—H2A	109.8
S1—Zn1—S3	116.788 (16)	C1—C2—H2A	109.8
S2—S1—Zn1	98.167 (18)	N2—C2—H2B	109.8
O2—S2—O3	112.85 (10)	C1—C2—H2B	109.8
O2—S2—O1	110.61 (9)	H2A—C2—H2B	108.3
O3—S2—O1	111.04 (10)	N2—C3—C4	109.77 (14)
O2—S2—S1	109.71 (6)	N2—C3—H3A	109.7
O3—S2—S1	107.00 (6)	C4—C3—H3A	109.7
O1—S2—S1	105.27 (6)	N2—C3—H3B	109.7
S4—S3—Zn1	101.05 (2)	C4—C3—H3B	109.7
O4—S4—O6	110.47 (8)	H3A—C3—H3B	108.2
O4—S4—O5	111.17 (9)	N1—C4—C3	113.09 (14)
O6—S4—O5	112.02 (9)	N1—C4—H4A	109.0
O4—S4—S3	110.45 (6)	C3—C4—H4A	109.0
O6—S4—S3	108.00 (6)	N1—C4—H4B	109.0
O5—S4—S3	104.53 (5)	C3—C4—H4B	109.0
C1—N1—C4	110.39 (12)	H4A—C4—H4B	107.8
C1—N1—Zn1	112.60 (10)	N3—C5—C6	113.06 (12)
C4—N1—Zn1	109.65 (9)	N3—C5—H5A	109.0
C1—N1—H1	105.4 (13)	C6—C5—H5A	109.0
C4—N1—H1	112.1 (13)	N3—C5—H5B	109.0
Zn1—N1—H1	106.6 (13)	C6—C5—H5B	109.0
C2—N2—C3	110.50 (14)	H5A—C5—H5B	107.8
C2—N2—H2AN	111.5 (13)	N4—C6—C5	110.10 (13)
C3—N2—H2AN	107.0 (14)	N4—C6—H6A	109.6
C2—N2—H2BN	107.5 (14)	C5—C6—H6A	109.6
C3—N2—H2BN	114.4 (14)	N4—C6—H6B	109.6
H2AN—N2—H2BN	106.1 (19)	C5—C6—H6B	109.6
C5—N3—C8	110.20 (12)	H6A—C6—H6B	108.2
C5—N3—Zn1	112.71 (9)	N4—C7—C8	110.19 (13)
C8—N3—Zn1	111.95 (9)	N4—C7—H7A	109.6
C5—N3—H3	109.2 (12)	C8—C7—H7A	109.6
C8—N3—H3	106.3 (12)	N4—C7—H7B	109.6
Zn1—N3—H3	106.2 (12)	C8—C7—H7B	109.6
C6—N4—C7	110.40 (12)	H7A—C7—H7B	108.1
C6—N4—H4AN	113.4 (13)	N3—C8—C7	112.29 (12)
C7—N4—H4AN	107.0 (13)	N3—C8—H8A	109.1
C6—N4—H4BN	114.3 (13)	C7—C8—H8A	109.1
C7—N4—H4BN	106.1 (13)	N3—C8—H8B	109.1
H4AN—N4—H4BN	105.1 (17)	C7—C8—H8B	109.1
N1—C1—C2	113.43 (15)	H8A—C8—H8B	107.9
N1—C1—H1A	108.9	H10A—O10—H10B	109 (3)
C2—C1—H1A	108.9	H20A—O20—H20B	100 (3)
			
C4—N1—C1—C2	52.0 (2)	C8—N3—C5—C6	−53.62 (19)
Zn1—N1—C1—C2	174.88 (12)	Zn1—N3—C5—C6	−179.47 (11)
C3—N2—C2—C1	59.1 (2)	C7—N4—C6—C5	−57.01 (18)
N1—C1—C2—N2	−56.1 (2)	N3—C5—C6—N4	55.66 (19)
C2—N2—C3—C4	−59.4 (2)	C6—N4—C7—C8	58.03 (18)
C1—N1—C4—C3	−51.76 (19)	C5—N3—C8—C7	54.16 (18)
Zn1—N1—C4—C3	−176.37 (11)	Zn1—N3—C8—C7	−179.56 (11)
N2—C3—C4—N1	56.0 (2)	N4—C7—C8—N3	−56.96 (19)

### Hydrogen-bond geometry (Å, º)

**Table:**

*D*—H···*A*	*D*—H	H···*A*	*D*···*A*	*D*—H···*A*
N1—H1···O2^i^	0.83 (2)	2.25 (2)	3.041 (2)	160 (2)
N2—H2*AN*···O6^ii^	0.95 (3)	1.80 (3)	2.730 (2)	166 (2)
N2—H2*BN*···O10	0.93 (2)	1.89 (2)	2.811 (3)	170 (2)
N3—H3···O3^i^	0.84 (2)	2.03 (2)	2.853 (2)	166 (2)
N4—H4*AN*···O20	0.87 (2)	2.09 (2)	2.892 (2)	154 (2)
N4—H4*BN*···O5^iii^	0.94 (2)	1.84 (2)	2.763 (2)	169 (2)
O10—H10*A*···O4	0.85 (3)	1.94 (4)	2.780 (2)	175 (3)
O10—H10*B*···O1^iv^	0.76 (3)	2.02 (3)	2.777 (2)	176 (4)
O20—H20*A*···O1^v^	0.82 (3)	2.02 (3)	2.804 (2)	163 (3)
O20—H20*B*···O5^vi^	0.74 (3)	2.17 (3)	2.866 (2)	158 (3)
C3—H3*A*···O6^iv^	0.97	2.45	3.221 (2)	136
C4—H4*A*···O3	0.97	2.49	3.175 (3)	128
C4—H4*B*···O4	0.97	2.54	3.463 (2)	159
C5—H5*B*···S3	0.97	2.86	3.453 (2)	120
C8—H8*B*···O2	0.97	2.50	3.318 (2)	142

Symmetry codes: (i) −*x*+1, −*y*+1, −*z*; (ii) *x*+1, *y*, *z*; (iii) *x*, *y*+1, *z*; (iv) −*x*+1, −*y*, −*z*; (v) −*x*, −*y*+1, −*z*; (vi) −*x*, −*y*+1, −*z*+1.
